# Brahma Rasayana enhances *in vivo* antioxidant status in cold-stressed chickens (*Gallus gallus domesticus*)

**DOI:** 10.4103/0253-7613.55209

**Published:** 2009-06

**Authors:** V. Ramnath, P.S. Rekha

**Affiliations:** Dept. of Veterinary Physiology, College of Veterinary and Animal Sciences, Kerala Agricultural University, Mannuthy, Thrissur-680 651, Kerala, India

**Keywords:** Antioxidants, brahma rasayana, chicken

## Abstract

**Objective:**

To evaluate the antioxidant status of chicken during cold stress and to investigate if there are any beneficial effects of Brahma Rasayana supplementation in cold stressed chicken.

**Materials and Methods:**

Activities of enzymatic and levels of non-enzymatic antioxidants in blood / serum and liver tissue were evaluated in chicken exposed to cold (4 ± 10C and relative humidity of 40 ± 5%, for six consecutive hours daily, for 5 or 10 days). The antioxidant properties of Brahma Rasayana (BR) supplementation (2 g/kg daily, orally) during cold stress was also studied.

**Results:**

There was a significant (*P* < 0.05) decrease in antioxidant enzyme in the blood, such as, superoxide dismutase (SOD), glutathione peroxidase (GPX), glutathione reductase (GR), and serum reduced glutathione (GSH) in cold stressed chicken. Serum and liver lipid peroxidation levels were significantly (*P* < 0.05) higher in cold stressed untreated chickens when compared to the treated and unstressed groups. There was also a significant (*P* < 0.05) increase in the antioxidant enzymes in the blood, such as, catalase (CAT) and SOD, in the liver CAT and SOD, and in GPX and GR in BR-treated cold stressed chicken, when compared to the untreated controls.

**Conclusions:**

Results of the present study conclude that in chicken, BR supplementation during cold stress brings about enhanced actions of the enzymatic and non-enzymatic antioxidants, which nullify the undesired side effects of free radicals generated during cold stress.

## Introduction

Stress is a state of threatened homeostasis provoked by psychological, physiological or environmental stress. A stressful condition leads to the excessive production of free radicals, which results in oxidative stress and an imbalance in the oxidant / antioxidant system.[1] During environmental stress, elevation in the corticosterone level accelerates the generation of free radicals and suppresses the immune function.[2] Antioxidants are effective in stressful conditions, as they readily give up their own electrons to free radicals.

Breeding hens are continuously exposed to oxidative stress over their lifetime, and the cumulative stress may cause diseases.[3] Hence, an effective antioxidant system for breeding pullets can be of great importance. Under routine production conditions various types of stress are experienced by chickens such as heat / cold, transport, preslaughter holding, ventilation, and so on. At temperatures above or below the thermo-neutral zone, corticosteroid secretion increases in response to stress.[4] By decreasing synthesis and secretion of corticosteroids, Vitamin C alleviates the negative side effects of stress. Although poultry can synthesize Vitamin C, it becomes inadequate under stressful conditions, such as, low or high environmental temperature, high humidity, high egg production rate, and parasite infestation.[5]

Brahma Rasayana (BR) is a nontoxic polyherbal preparation, derived from plant extracts, and is claimed to be a potent immunomodulator in cancer patients undergoing chemotherapy and radiation therapy.[6] Gooseberry (*Emblica officinalis*) and Indian gall nut (*Terminalia chebula*) are the two major ingredients of BR accounting for more than 75% *w/w* of the preparation.[7] There are studies indicating that the antioxidant activities of gooseberry are attributed to ascorbic acid and polyphenols, such as, ellagic acid, gallic acid, tannins and so on. Earlier studies revealed that BR is a potent oxygen-free radical scavenger of *in vitro* and *in vivo* models.[8]

The objective of the study was to evaluate the antioxidant properties of BR by measuring the lipid peroxidation (LPO), enzymatic superoxide dismutase (SOD), catalase (CAT), glutathione peroxidase (GPX), glutathione reductase (GR), and non-enzymatic reduced glutathione (GSH) content in chickens that are exposed to cold stress, and thereby assess the benefits of BR supplementation in achieving cold tolerance in chicken.

## Materials and Methods

### Birds

A total of 48 one day old male egg type domestic chickens (*Gallus gallus domesticus*) were procured from the University poultry farm, at the College of Veterinary and Animal Sciences, Kerala Agricultural University, Mannuthy, and reared in a battery brooder. They were fed with commercial starter feed for the first four weeks of their life. At the end of their brooding life, the chicks were fed with commercial adult layer mash, till they attained 1 kg body weight. The body weight was recorded weekly from the day of hatch up until the experiment's completion. Before commencing the work, permission from the Institutional Animal Ethics Committee was obtained.

### Brahma Rasayana

Brahma Rasayana is a mixture of *Emblica officinalis* (20%), *Terminalia chebula* (6.67%), *Urarira pitca* (0.4%), *Desmodium gangeticum* (0.4%), *Gmelina arborea* (0.4%), *Solanum nigrum* (0.4%), *Tribulus terrestris* (0.4%), *Aegle marmelos* (0.4%), *Premna tomentosa* (0.4%), *Stereospermum suvaeolens* (0.4%), *Sida rhombilfolia* (0.4%), *Boerhaavia diffusa* (0.4%), *Ricinus communis* (0.4%), *Vigna vexilata* (0.4%), *Phaseolus adenanthus* (0.4%), *Asparagus racemosus* (0.4%), *Holostemma annulare* (0.4%), *Leptadenia reticulate* (0.4%), *Desmostachya bipinnata* (0.4%),*Saccharum officinarum* (0.4%), *Oryza malampuzhensis* (0.4%), *Cinnamomum iners* (0.16%), *Elettaria cardamomum* (0.16%), *Cyperus rotundus* (0.16%), *Curcuma longa* (0.16%), *Piper longum* (0.16%), *Aquilaria agallocha* (0.16%), *Santalum album* (0.16%), *Centella asciatica* (0.16%), *Mesua ferrea* (0.16%), *Clitoria ternate* (0.16%), *Acorus calamus* (0.16%), *Scirpus crossus* (0.16%), *Glycyrrhiza glabra* (0.16%), and *Embelia ribes* (0.16%).

Brahma Rasayana was prepared as per standard recommended procedures,[9] and purchased from Vaidyaratnam Oushadhasala, Ollur, Thrissur, Kerala.

### Administration of BR

The required quantity of BR was made into suspension using warm water (five parts) and mixed with about 30 g of poultry feed (one-third of the total daily ration) and fed to the experimental birds daily, as the first meal. The oral administration of BR at various dose levels, ranging from 0.5 to 6.0 g/kg b.wt., of the bird, daily, for 20 days, was tried, and it did not induce any drug-related toxicity. No significant weight loss or gain was observed, reflecting the effect of BR administration on the well-being of birds. Even at the highest concentration of 6.0 g/kg b.wt., the birds showed no visible toxicity as measured by loss of appetite, lack of movement or alertness. Therefore, it was concluded that BR was nontoxic. However, for all experiments the dose used was 2.0 g/kg b.w, which was at par with the dose administered to mice, that is, for 50 mg of mouse 25 g.wt.[8]

### Cold stress regime

The protocol followed in this study was a combination of two earlier reports.[10,11] A controlled environmental unit with a holding capacity of 12 birds at a time, in six chambers (area 1800 cm^2^ / chamber), with provisions for adjusting humidity and temperature was used. The chamber temperature of 4 ± 1°C and relative humidity (RH) of 40 ± 5% was chosen for the present study. Chickens were exposed to the above conditions in the chamber, for six consecutive hours / day for five or 10 days. During the period of cold stress in the chamber, the chickens were neither provided with drinking water nor feed.

### Experimental design

Group I: Non-cold stressed (NCST) and untreated birds, reared randomly under an ambient temperature of 30 ± 1°C and 65% RH.

Group II: NCST and BR treated (2 g/kg b.wt. p.o.) for 20 days, reared under similar conditions of temperature and humidity as Group I.

Group III: Cold stressed (CST) at 4 ± 1°C and RH of 40 ± 5% for six consecutive hours / day for five days.

Group IV: CST at 4 ± 1°C and RH of 40 ± 5% for six consecutive hours / day for five days and BR treated (2 g/kg b p.o.) for 15 days (10 days prior to and five days during the experimental period of cold exposure).

Group V: CST at 4 ± 1°C and RH of 40 ± 5% for six consecutive hours /day for 10 days.

Group VI: CST at 4 ± 1°C and RH of 40 ± 5% for six consecutive hours /day for five days and BR treated (2 g/kg b. p.o.) for 20 days (10 days prior to and 10 days during the experimental period of cold exposure).

### Blood and tissue collections from birds

Blood samples (3 ml) were collected from the wing vein of birds with and without anticoagulant (heparin) from groups III and V after five days of CST, and from birds of groups IV and VI after 10 days of CST. Blood collection was done on day 20, from all birds of group I and II controls. Immediately after collection, the serum was separated, aliquoted, and used for assays. On the same day of blood collection, the birds were sacrificed by cervical dislocation. The liver was excised and thoroughly washed in ice cold saline (0.9%). It was perfused with ice cold saline via the portal vein before homogenization.[12] A liver homogenate (10%) was made in an ice cold Tris-HCl buffer (pH 7.4) and a cytosolic sample of liver homogenate was obtained by centrifuging at 10,000 g for 30 minutes, at 4°C.

### Biochemical analysis

The catalase (CAT) activity in fresh blood (erythrocytes) and liver was assessed by the decomposition of hydrogen peroxide, according to Aebi's method and the activities expressed as Kat/g Hb and Units /mg protein.[13] The superoxide dismutase (SOD) activity in fresh blood (erythrocytes) and liver was determined by the photoreducion of nitroblue tetrazolium (NBT), according to the McCord and Fridovich method.[14] One unit of SOD activity was defined as the amount of protein that inhibited the rate of reduction of NBT by 50% and was expressed as Units/g Hb and Units/mg protein. Serum- and liver-reduced glutathione (GSH) content were determined by their reaction with 5-5'dithiobis (2-nitrobenzoic acid), according to Moron *et al*., and the concentration in the serum and liver was expressed as nmol/ml and nmol/mg protein.[15] Liver Glutathione peroxidase (GPx) activity was assessed by degradation of hydrogen peroxide (H_2_O_2_) in the presence of reduced glutathione, according to Paglia and Valentine's method, and the activity expressed as Units/mg protein.[16] Liver cytosolic glutathione reductase (GR) activity was determined by the amount of NADPH consumed in the conversion of oxidized glutathione (GSSG) to GSH, by following the Racker's method and the activity expressed as nmol of NADPH consumed/min/mg protein.[17] Serum and liver lipid peroxidation levels were assessed by determining small amounts of malondialdehyde (MDA) produced during peroxidation, following Ohkawa *et al*., and the concentration in serum and liver was expressed as nmol of MDA formed per ml and per mg protein.[12] Protein concentration in the liver was estimated by Lowry's method and expressed in terms of mg protein.[18]

### Statistical analysis

Data were expressed as mean ± standard error (SE). Statistical analysis was done by one-way classification of ANOVA followed by Duncan's Multiple Range Test (DMRT).[19] *P* < 0.05 was considered as significant.

## Results

### Cold stress influenced levels of blood antioxidant enzymes

Untreated CST birds of Group III and V exhibited significantly (*P* < 0.05) lower activity of blood erythrocyte CAT (22.00 ± 0.57 K/g Hb and 24.63± 0.87 K/g Hb, respectively) [Table 1] and blood SOD (236.50 ±1.65 U/g Hg and 246.63 ±3.41 U/g Hg, respectively) [Table 1] when compared with NCST – BR treated controls of Group II birds (100.25 ± 1.05 K/g Hb and 362.13 ± 2.46 U/g Hg, respectively).

**Table 1:** Effect of cold stress on activities of blood catalase (CAT), superoxide dismutase (SOD), serum content of reduced glutathione (GSH), and serum lipid peroxidation level in egg type male chicken

*Groups*	*Blood*		*Serum*	
		
	*CAT (K/g Hb)*	*SOD (U/g Hb)*	*GSH content (nmol/ml)*	*Lipid peroxidation level (nmol of MDA formed/ml)*
I	24.04de ± 0.31	294.63d ± 3.68	144.88d ± 1.82	2.39d ± 0.03
II	100.24a ± 1.05	362.13c ± 2.46	176.20b ± 0.45	2.42d ± 0.02
III	22.00e ± 0.57	236.50e ± 1.65	157.38c ± 1.80	5.94b ± 0.14
IV	39.13b ± 1.29	384.50b ± 7.92	216.63a ± 8.29	4.50c ± 0.11
V	24.63d ± 0.87	246.63e ± 3.41	72.38f ± 1.64	6.40a ± 0.08
VI	33.25c ± 0.77	409.63a ± 1.93	92.75e ± 2.03	2.58d ± 0.13

Data are means ± SEM. Used ANOVA followed Duncan's Multiple Range Test. Means in columns having different superscript/s (a to f) differ significantly between groups at 5% level

### Untreated birds showed increase in non-enzymatic antioxidants during cold stress

Untreated CST birds in group III and V exhibited significantly (*P* < 0.05) lower levels of serum GSH (157.38 ± 1.80 nmol/ml and 72.38 ± 1.64 nmol/ml, respectively)

[Table 1] when compared to NCST – BR treated controls of Group II birds (176.25^b^ ± 0.45 nmol/ml). However, the liver GSH level of untreated birds belonging to group III and V exhibited significantly (*P* < 0.05) higher activity (2.04 ± 0.01 and 2.47 ± 0.03, respectively) when compared to NCST – untreated controls of Group I birds (1.53± 0.03) [Table 2].

**Table 2:** Effect of cold stress on activities of liver catalase (CAT), superoxide dismutase (SOD), glutathione peroxidase (GPX), glutathione reductase (GR), reduced glutathione (GSH) content, and lipid peroxidation level in egg type male chicken

*Groups*	*CAT*	*SOD*	*GPX*	*GR*	*GSH content*	*Lipid peroxidation level*
I	23.19^c^ ± 0.29	132.88^d^ ± 1.07	9.20^c^ ± 0.03	504.75^c^ ± 2.0	1.53^d^ ± 0.03	0.50^bc^ ± 0.02
II	35.90^b^ ± 0.42	184.00^b^ ± 0.80	12.65^a^ ± 0.10	1055.88^a^ ± 3.07	2.28^b^ ± 0.02	0.57^b^ ± 0.02
III	34.43^b^ ± 0.42	169.25^c^ ± 1.37	6.40^d^ ± 0.09	237.13^f^ ± 2.0	2.04^c^ ± 0.10	1.01^a^ ± 0.04
IV	60.86^a^ ± 0.74	189.88^ab^ ±3.09	9.23^c^ ± 0.06	368.00^d^ ± 2.2	2.02^c^ ± 0.04	0.45^c^ ± 0.02
V	19.24^d^ ± 0.52	170.88^c^ ± 2.15	9.80^b^ ± 0.20	311.00^e^ ± 2.32	2.47^b^ ± 0.03	1.04^a^ ± 0.05
VI	62.24^a^ ± 0.65	192.13^a^ ± 4.08	9.99^b^ ± 0.15	699.63^b^ ± 8.29	2.32^b^ ± 0.05	0.43^c^ ± 0.02

Data are means ± SEM. Used ANOVA followed Duncan's Multiple Range Test. Means in columns having different superscript/s (a to f) differ significantly between groups at 5% level

### Damage to the liver in cold stress was averted in BR treated birds

Untreated CST birds in group III and V exhibited a significant (*P* < 0.05) increase in activities of liver SOD when compared to NCST – untreated controls of Group I birds [Table 2]. However there was significant (*P* < 0.05) decrease in liver CAT activity in group V birds when compared to NCST – untreated controls of Group I birds [Table 2]. A similar kind of decrease in liver GPX activity was encountered in group III birds when compared to NCST – untreated controls [Table 2]. Untreated CST birds in group III and V exhibited significantly (*P* < 0.05) lower activity of liver GR when compared to NCST – untreated controls of Group I birds [Table 2].

### BR improved enzymatic and non-enzymatic antioxidants in blood and liver

When compared to untreated CST birds (Group III and V), BR treated birds of NCST and CST groups (groups II, IV, and VI) exhibited significantly (*P* < 0.05) increased activities of erythrocyte CAT (10 to 50% increase) [Table 1], erythrocyte SOD (40 to 60% increase) [Table 1], and levels of serum GSH [Table 1]. However, in the liver tissues, no such significant differences were encountered between NCST / CST – BR treated (group II vs. VI) and CST – untreated / BR – treated (group III vs. IV) groups [Table 2] with regard to GSH level. Similarly, significantly (*P* < 0.05) higher activity of all liver antioxidant enzymes, such as, CAT, SOD, and GR except GPX was observed in NCST / CST – BR treated (groups II, IV, and VI) when compared to NCST / CST – untreated birds (groups I, III, and V) [Table 2].

### Lipid peroxidation (LPO) levels in serum and liver increased in untreated CST birds

All untreated CST birds of group II and V showed significant (*P* < 0.05) increase in LPO levels in the serum (5.94 ± 0.14 nmol of MDA formed / ml and 6.40 ± 0.08 nmol of MDA formed / ml, respectively) [Table 1] and liver (1.01 ± 0.04 nmol of MDA formed / mg protein and 1.04 ± 0.05 nmol of MDA formed / mg protein, respectively) [Table 2] when compared to the NCST – untreated control birds of group I (2.39 ± 0.03 nmol of MDA formed / ml of serum and 0.50 ± 0.02 nmol of MDA formed / mg protein of liver, respectively) [Tables 1 and 2]. There was a significant (*P* < 0.05) decrease in the serum LPO levels of CST – BR treated birds of group IV and VI (4.50 ± 0.11 nmol of MDA formed / ml and 2.58 ± 0.13 nmol of MDA formed / ml, respectively) observed when compared to the CST – untreated birds of group III and V [Table 1]. A significant (*P* < 0.05) decrease in the LPO levels of the liver tissue of CST – BR treated birds of group IV and VI (0.45 ± 0.02 nmol of MDA formed / mg protein and 0.43 ± 0.02 nmol of MDA formed / mg protein, respectively) was observed when compared to the CST – untreated birds of group III and V [Table 2].

## Discussion

Environmental stress causes oxidative stress *in vivo* resulting in an imbalance in the antioxidant status due to excessive formation of free radicals like peroxides, singlet oxygen, and free radicals.[20] Lower levels of plasma antioxidant such as vitamin C, vitamin E, zinc and chromium, and increased oxidative damage, were observed in stressed poultry.[21,22]

Exposure to cold is a direct threat to the body. When the surrounding temperature drops below 8°C, the body may not be able to warm itself, and hence, serious cold-related illnesses, permanent tissue damage, and even death may result.[23] Effective *in vivo* antioxidant mechanisms include vitamins A, C, and E, glutathione, and antioxidant enzymes, are essential for protecting the organism against damage resulting from ROS.[24] Rich source of gallic acid is found in *Terminalia chebula* and vitamin C in *Emblica officinalis*. The endogenous antioxidant system counteracts the ROS and reduces the oxidative stress. Vitamin C, well known as a potent water soluble, non-enzymatic antioxidant effectively intercept oxidants in the aqueous phase before they attack and cause detectable oxidative damage.[25] Reduced glutathione is considered to be the master antioxidant of the body and is found in almost all living cells. The body utilizes GSH chiefly for reducing oxidized vitamin C and E back to their reduced state, to detoxify many toxins, to maintain the cellular redox potential, to maintain erythrocyte membrane integrity and so on. Glutathione peroxidase (GPx) and glutathione reductase (GR) are enzymes associated with conversion of GSH to the oxidized form and back.[26] The liver is the chief organ concerned with the detoxification process and availability of an increased amount of GSH in the liver ensures this function. Depletion in the activity of these enzymatic and non-enzymatic antioxidants can be the result of enhanced radical production during stress conditions.

In this study it was found that untreated birds exposed to cold for five or 10 days invariably showed reduced activities of blood CAT and SOD as well as serum GSH levels when compared to both control and CST – BR treated birds. Earlier reports supported the present findings that BR supplementation alone at 2 g/kg b.wt. p.o. for 20 days in NCST birds (group II) facilitated an increased activity of CAT and SOD.[25] This could be beneficial for the birds, as increased antioxidant activity in erythrocytes ensured proper and rapid elimination of ROS that could be formed during cold stress, thereby protecting the integrity of the erythrocyte membrane. The BR supplementation to CST / NCST birds resulted in increased serum GSH content, when compared to their untreated counterparts.

Results, herein, revealed that the GSH content in the liver of NCST / CST – BR treated birds became significantly elevated over the NCST untreated group, concomitant with activities of GPx and GR enzymes. The rate of GSH formation and the rate at which it was oxidized by antioxidant liver enzymes did not match that in the CST – untreated birds, while in NCST / CST – BR treated birds an appreciable balance between activities of oxidizing and reducing enzymes of glutathione was observed, at least by day 10 of the cold treatment. Thus it could be considered as one of the markers of cold tolerance / acclimatization.

Lipid peroxidation is regarded as one of the basic mechanisms of tissue damage caused by free radicals, as observed in aging, atherosclerosis, and other pathological disorders. LPO of serum lipids, especially that of HDL is highly unfavorable, which may result in many vascular disorders. Results of this study revealed that LPO levels in the serum and liver of NCST / CST – BR treated birds were significantly lower than those encountered in CST – untreated birds. These results are suggestive of the beneficial aspects of BR supplementation, 10 days prior to CST, to be continued during the period of CST in birds; thereby, successfully neutralizing the undesired side effects produced by the free radicals that were generated.

On account of the increased activity and concentration of enzymatic and non-enzymatic antioxidants that resulted from oral BR supplementation, the birds remained healthy and maintained body weight. It may be concluded that BR treatment facilitates earlier adaptation of chickens to stressful cold conditions.
